# Reduced-Order Distributed Unknown Input Observer for State Estimation and Unknown Input Reconstruction

**DOI:** 10.3390/s26041307

**Published:** 2026-02-18

**Authors:** Shuqing Tang, Zongjun Zhu, Xufeng Ling, Fanglai Zhu

**Affiliations:** 1 Shanghai Fiswft Intelligent Technology Co., Ltd., Shanghai 201800, China; shq.tang95@gmail.com; 2 College of Electronics and Information Engineering, Tongji University, Shanghai 201804, China; zhuzongjun@tongji.edu.cn; 3 School of Artificial Intelligence, Shanghai Normal University Tianhua College, Shanghai 201815, China; lxf1131@sthu.edu.cn

**Keywords:** distributed unknown input observer, interval observer, distributed reduced-order observer, measurement noise reconstruction, unknown input reconstruction

## Abstract

In traditional observer designs, simultaneous state estimation, unknown input reconstruction (UIR), and measurement noise reconstruction have been fully discussed. However, in distributed observer, this remains to be investigated, and few papers deal with this problem. This paper designs a reduced-order distributed unknown input observer (DUIO) to offer the asymptotic convergent estimations of both the states, the unknown input (UI) and measurement noise (MN), for the uncertain target system with UI and each sensor node with MN. Firstly, by introducing an auxiliary output, the local system in every node is transformed into a system whose output contains no MN. Secondly, for the transformed local system, a distributed reduced-order observer produces asymptotically convergent state estimations. Thirdly, based on the interval observer and state estimation, a UIR is designed, and the UIR can estimate the real UI asymptotically. Moreover, the UIR decouples the control input. Finally, a simulation example is given to illustrate the effectiveness and advantages of the proposed method.

## 1. Introduction

With the rise of multi-agent cooperative technology, the distributed estimation problem has gradually attracted researchers’ interests [1,2,3,4,5,6,7]. In distributed estimation problems, a group of sensor nodes is deployed to form distributed observers that collaboratively estimate the state of the target system. Typically, each node possesses measurement, computation, and communication capabilities [8,9]. The advantages of distributed observers lie in their greater cost-effectiveness, easier scalability and maintenance, and enhanced robustness.

In the design of distributed observers, due to limited information acquisition, a single sensor node cannot independently reconstruct the state. Therefore, it must communicate with neighboring nodes, meaning that cooperation among nodes is essential to address the distributed estimation problem. For example, ref. [10] investigated the distributed observer problem, where the information exchange among nodes forms the communication topology, and employed the node-wise detectability decomposition approach to simplify the design of distributed observers.

Recently, event trigger mechanism, jointly switching topology, and UI have been considered in distributed state estimations, and many significant results have been reported in the literature [11,12,13,14,15,16,17,18,19,20,21]. For example, ref. [16] investigates the distributed estimation problem under scenarios where communication channels are subjected to stochastic impulsive attacks. However, until recently, only a few studies have taken external disturbance and measurement noise into consideration. Refs. [22,23,24] consider the distributed observation problem when the system is subject to the external UI without providing the reconstruction of the external UI. The interval observer, an important technique for estimating the stable upper and lower boundaries of the system states based on the upper and lower boundaries of UI, has received extensive attention as shown in [25,26,27,28,29,30,31,32,33]. For example, refs. [28,29] design a distributed interval unknown input observer that is able to obtain simultaneously interval state observation and UI estimation. The studies in [30,31] develop distributed interval observer frameworks to systematically handle state interval observations for the systems under simultaneous external disturbances and MN. Although significant progress has been made in existing work (particularly in interval observers and distributed UI observers [28,29,30,31,34]), in a distributed framework, achieving simultaneous, asymptotic estimation of the state, unknown inputs, and measurement noise remains an open challenge.

Classical unknown input observers (UIOs) provide a precise approach to resist disturbances. For example, ref. [35] discuss the state estimation for the uncertain systems. Refs. [36,37,38,39,40] not only consider the state observation but also the estimation of the UI, which attracts an interest in DUIO. Guided by the idea in the classical UIO design, this article proposes a reduced-order DUIO, which combines a distributed reduced-order observer and a local interval observer. The core contributions of this article are listed as follows.

(i) An output transformation is applied to remove the measurement noise, resulting in an MN-free output. In order to handle the UI, a state transformation is taken, resulting in the state equation being divided into parts, where one part decouples the UI.

(ii) A reduced-order DUIO design method is developed by first constructing a distributed observer from the UI-free state equation to attain asymptotic distributed state estimation.

(iii) By employing an interval observer to obtain bounded output estimates, an algebraic relationship linking the UI to the state can thus be constructed. The UIR can estimate the actual UI asymptotically, and, moreover, it decouples the control input. Thus will benefit in designing the controller to compensate for the influence of the input.

## 2. Preliminaries

### 2.1. Notations

$\ker\left(G\right)$ and $\mathrm{im}\left(G\right)$ denote, respectively, the kernel space and image space of matrix *G*. When *G* is a square matrix, $\lambda_{\min}\left(G\right)$ and $\lambda_{\max}\left(G\right)$ denote, respectively, the minimal and maximum eigenvalues of *G*. For matrices $G_{i}\in R^{r_{i}\times s_{i}}$, ${\mathrm{diag}}_{i\in N}\left(G_{i}\right)\in R^{r\times s}$ stands for block diagonal matrix with $G_{i}$ being its diagonal matrices, where $N=\left\{1,\ldots ,N\right\}$, $r=r_{1}+\cdots +r_{N}$ and $s=s_{1}+\cdots +s_{N}$. For a vector $g\in R^{n}$, $\mathrm{diag}\left(g\right)\in R^{n\times n}$ is a diagonal matrix and its diagonal elements are just the elements of the vector. For two matrices or vectors $U=\left[u_{ij}\right]\in R^{m\times n}$ and $V=\left[v_{ij}\right]\in R^{m\times n}$, U≤V⇔$u_{ij}\leq v_{ij}$ (i=1,…,m;j=1,…,n). Let $1_{N}$ be a column vector with elements being 1s dimension being *N*. $∥\cdot ∥$ means the Euclidean norm of a corresponding vector. Denote the Kronecker product as ⊗. The vector ${\left[x_{1}^{T},\ldots ,x_{n}^{T}\right]}^{T}$ is denoted by $\mathrm{col}\left(x_{1},\ldots ,x_{n}\right)$. For a matrix $W=\left[w_{ij}\right]\in R^{N\times N}$, denote $w_{ij}^{+}=\max\left(0,w_{ij}\right)$ and $w_{ij}^{-}=\max\left(0,-w_{ij}\right)$, and then define $W^{+}=\left[w_{ij}^{+}\right]$, $W^{-}=\left[w_{ij}^{-}\right]$ and $\left|W\right|=\left[\left|w_{ij}\right|\right]$. Then, we have $W=W^{+}-W^{-}$ and $\left|W\right|=W^{+}+W^{-}$.

### 2.2. Basic Graph Theory

The information communication among nodes can be shown by a graph $G=\left(V,E,A\right)$, where $V=\left\{1,2,\ldots ,N\right\}$ represents the node set, E⊆V×V represents the edge set, and $A=\left[a_{ij}\right]\in R^{N\times N}$ is the adjacent matrix. (i,j)∈E means that node *i* can directly communicate with node *j*. We always assume that $a_{ii}=0$, and, additionally, $a_{ij}>0$ when (i,j)∈E; otherwise, $a_{ij}=0$. Denote the Laplacian matrix as $L={\left[l_{ij}\right]}_{N\times N}\in R^{N\times N}$, where $l_{ij}=-a_{ij}$, when i≠j and $l_{ii}=\sum_{k=1}^{N}a_{ik},\;\left(i=1,\ldots ,N\right)$. For all *i* and *j*, graph G is named an undirected graph, when $\left(i,j\right)\in E$ if and only if $\left(j,i\right)\in E$.

**Definition** **1.** *There exists a path from node i to j, if the exists a series of edges: $\left(i,i_{1}\right)\in E$, $\left(i_{1},i_{2}\right)\in E$, ⋯ , $\left(i_{l-1},i_{l}\right)\in E$, $\left(i_{l},j\right)\in E$, where $i_{k}\in V$ (k=1,…,l). A directed graph is said to be strongly connected if there exists a path from any node to any other node.*

**Lemma** **1** ([10]). *If the graph G is strongly connected, there must exists a vector $\theta =\mathrm{col}\left(\theta_{1},\ldots ,\theta_{N}\right)$, such that $\sum_{i=1}^{N}\theta_{i}=1$, $\theta^{T}L=0$ and $\theta_{i}>0$. $\hat{L}=\Theta L+L^{T}\Theta$ is a symmetric positive definite matrix, where $\Theta ={diag}_{i\in N}\left(\theta_{i}\right)$.*

**Definition** **2.** *For two matrices $P\in R^{n\times n}$ and $H\in R^{p\times n}$, the unobservable subspace of the pair $\left(H,P\right)$ is defined by $UO\left(H,P\right)={⋂}_{j=1}^{n}\ker\left(HP^{j-1}\right)$, and the undetectable subspace is $UD\left(H,P\right)=UO\left(H,P\right)⋂\left[\ker\left(\alpha_{P}^{+}\left(P\right)\right)\right]$, where $\alpha_{P}\left(s\right)=\alpha_{P}^{+}\left(s\right)\alpha_{P}^{-}\left(s\right)$. Here $\alpha_{P}\left(s\right)$ stands for the characteristic polynomial of the matrix, $\alpha_{P}^{+}\left(s\right)$ is the polynomial with roots of zero or positive real parts and $\alpha_{P}^{-}\left(s\right)$ is the one with roots of negative real parts.*

### 2.3. System Description

Considering the following multiple sensor system with *N* sensors

(1) $$\begin{array}{c} \begin{array}{cc} \dot{x}\left(t\right) & =Ax\left(t\right)+Bu\left(t\right)+Df\left(t\right)\\ y_{i}\left(t\right) & =C_{i}x\left(t\right)+W_{i}\omega_{i}\left(t\right),\left(i\in N=\left\{1,\cdots ,N\right\}\right) \end{array} \end{array}$$

where $x\in R^{n}$ is the state, $u\in R^{m}$ is the input, and $f\in R^{q}$ is the UI. Additionally, $y_{i}\in R^{p_{i}}$ is the output vector of the *i*th sensor, while $\omega_{i}\in R^{g_{i}}$ is the MN affecting the *i*th sensor. $A\in R^{n\times n}$, $B\in R^{n\times m}$, $D\in R^{n\times q}$, $C_{i}\in R^{p_{i}\times n}$ and $W_{i}\in R^{p_{i}\times g_{i}}$ are all known constant matrices. Assume that $C_{i}$ is with full row rank, *D* and $F_{i}$ are of full column ranks. Moreover, introduce notations of $C=\mathrm{col}\left(C_{1},\cdots ,C_{N}\right)$$\in R^{p}$ and $W=\mathrm{diag}\left(W_{i}\right)\in R^{p\times g}$, where $p=\sum_{i=1}^{N}p_{i}$ and $g=\sum_{i=1}^{N}g_{i}$.

**Assumption** **1.** *The condition*

(2) $$\begin{array}{c} \mathrm{rank}\left[\begin{array}{ccc} sI_{n}-A & D & 0\\ C & 0 & W \end{array}\right]=n+q+g \end{array}$$

*holds for all complex s with Re(s)>0*

**Assumption** **2.** *The condition*

$$\begin{array}{c} \mathrm{rank}\left[\begin{array}{ccc} I_{n} & D & 0\\ C_{i} & 0 & W_{i} \end{array}\right]=n+q+g_{i}\left(i\in N\right) \end{array}$$

*holds.*

**Remark** **1.** *Assumption 2 is the so-called observer matching condition, which is the necessary condition of designing the UIO.*

For system (1), a total of *N* sensors are deployed, and every sensor node can only access the partial output $y_{i}$, which suffers from the measurement noise $\omega_{i}$. The problem to be solved in this paper is to design a DUIO such that, in every sensor node, the state observation ${\hat{x}}_{i}$, the UIR ${\hat{f}}_{i}$, and measurement noise reconstruction ${\hat{\omega }}_{i}$ can be obtained. That is, $\lim_{t\to \infty }{\hat{x}}_{i}\left(t\right)-x\left(t\right)=0$, $\lim_{t\to \infty }{\hat{f}}_{i}\left(t\right)-f\left(t\right)=0$ and $\lim_{t\to \infty }{\hat{\omega }}_{i}\left(t\right)-\omega \left(t\right)=0$.

## 3. DUIO Design

In this section, a DUIO which consist of a distributed reduced-order observer and UIR is given. The distributed reduced-order observer is designed to estimate the system states and MN. Based on the interval observer and state estimation, a UIR is given.

### 3.1. Distributed Reduced-Order Observer Design

For system (1), take an output transformation $y_{a,i}=\left(I_{p_{i}}-W_{i}W_{i}^{†}\right)y_{i}$; then, $y_{a,i}=C_{a,i}x$, where $C_{a,i}=\left(I_{p_{i}}-W_{i}W_{i}^{†}\right)C_{i}$. Here $W_{i}^{†}$ is the pseudo inverse matrix of $W_{i}$ satisfying $W_{i}W_{i}^{†}W_{i}=W_{i}$.

**Lemma** **2.** *$\mathrm{rank}\left(C_{a,i}\right)=p_{i}-g_{i}:={\tilde{p}}_{i}$ implies that the matrix $C_{a,i}$ is with not full row rank when the ith sensor is subject to measurement noise.*

**Proof.**  Given that the matrix $W_{i}$ is full column rank, its singular value decomposition yields orthogonal matrices $Y\in R^{p_{i}\times p_{i}}$ and $Z\in R^{g_{i}\times g_{i}}$, satisfying $W_{i}=Y\left[\begin{array}{c} \Sigma \\ 0_{{\tilde{p}}_{i}\times g_{i}} \end{array}\right]Z^{T}$, in which $\Sigma =\mathrm{diag}(\sigma_{1},\ldots ,\sigma_{g_{i}})$ consists of the singular values of $W_{i}$. Furthermore, from the definition of the pseudo inverse $W_{i}^{†}={\left(W_{i}^{T}W_{i}\right)}^{-1}W_{i}^{T}$, it follows that $W_{i}^{†}=Z\left[\begin{array}{cc} \Sigma^{-1} & 0_{g_{i}\times {\tilde{p}}_{i}} \end{array}\right]Y^{T}$, and consequently $W_{i}W_{i}^{†}=Y\left[\begin{array}{cc} I_{g_{i}} & 0_{g_{i}\times \tilde{p}i}\\ 0\tilde{p}i\times g_{i} & 0{\tilde{p}}_{i}\times {\tilde{p}}_{i} \end{array}\right]Y^{T}$. Thus,

$$\begin{array}{c} \begin{array}{cc} C_{a,i} & =\left(I_{p_{i}}-W_{i}W_{i}^{†}\right)C_{i}\\ & =\left(YY^{T}-Y\left[\begin{array}{cc} I_{g_{i}} & \\ & 0_{{\tilde{p}}_{i}\times {\tilde{p}}_{i}} \end{array}\right]Y^{T}\right)C_{i}\\ & =Y\left[\begin{array}{cc} 0_{g_{i}\times g_{i}} & \\ & I_{{\tilde{p}}_{i}} \end{array}\right]Y^{T}C_{i} \end{array} \end{array}$$

yielding $\mathrm{rank}\left(C_{a,i}\right)={\tilde{p}}_{i}$.    □

Since $C_{a,i}$ is not full row rank, there exists an invertible matrix $\Upsilon_{i}\in R^{p_{i}\times p_{i}}$ such that $\Upsilon_{i}C_{a,i}=\left[\begin{array}{c} {\tilde{C}}_{i}\\ 0_{g_{i}\times n} \end{array}\right]$, where ${\tilde{C}}_{i}\in R^{{\tilde{p}}_{i}\times n}$ is composed of a maximal set of linearly independent rows from $C_{a,i}$. Consequently, $\mathrm{rank}\left({\tilde{C}}_{i}\right)={\tilde{p}}_{i}$, meaning ${\tilde{C}}_{i}$ is a full row rank matrix. It follows directly that ${\tilde{C}}_{i}=\left[\begin{array}{cc} I_{{\tilde{p}}_{i}} & 0 \end{array}\right]\Upsilon_{i}C_{a,i}=\Phi_{i}C_{i},$ where $\Phi_{i}=\left[\begin{array}{cc} I_{{\tilde{p}}_{i}} & 0 \end{array}\right]\Upsilon_{i}\left(I_{p_{i}}-W_{i}W_{i}^{†}\right)\in R^{g_{i}\times g_{i}}$.

An additional auxiliary output is introduced as ${\tilde{y}}_{i}=\left[\begin{array}{cc} I_{{\tilde{p}}_{i}} & 0 \end{array}\right]\Upsilon_{i}y_{a,i}=\Phi_{i}y_{i}$, then ${\tilde{y}}_{i}={\tilde{C}}_{i}x$. Thus, we can express system (1) as

(3) $$\begin{array}{c} \begin{array}{cc} \dot{x} & =Ax+Bu+Df\\ {\tilde{y}}_{i} & ={\tilde{C}}_{i}x\left(i\in N=\left\{1,\cdots ,N\right\}\right) \end{array} \end{array}$$

where ${\tilde{C}}_{i}$ is of full row rank.

**Lemma** **3.** *Given Assumption 1, there exists at least one complex $s_{i,0}$ with Re(si,0)≥0 such that*

(4) $$\begin{array}{c} \mathrm{rank}\left[\begin{array}{ccc} s_{i,0}I_{n}-A & D & 0\\ C_{a,i} & 0 & W_{i} \end{array}\right]<n+q+g_{i} \end{array}$$

*which also implies that the pair $\left(A,C_{a,i}\right)$ and thus the pair $\left(A,{\tilde{C}}_{i}\right)$ is undetectable.*

**Proof.**  Under Assumption 1, we know that there exists at least one complex $s_{0}$ satisfying Re(s0)>0, such that

$$\begin{array}{cc} \; & n+q+g=\mathrm{rank}\left[\begin{array}{ccc} s_{0}I_{n}-A & D & 0\\ C & 0 & W \end{array}\right]\\ & =\mathrm{rank}\left[\begin{array}{c} s_{0}I_{n}-A\\ C \end{array}\right]+q+g\\ \; & =\mathrm{rank}\left[\begin{array}{c} s_{0}I_{n}-A\\ C_{1}\\ \vdots \\ C_{N} \end{array}\right]+q+g\\ & <\mathrm{rank}\left[\begin{array}{c} s_{0}I_{n}-A\\ C_{i} \end{array}\right]+q+g\\ \; & =\mathrm{rank}\left(\left[\begin{array}{cc} I_{n} & 0\\ 0 & \Upsilon_{i}\left(I_{p_{i}}-W_{i}W_{i}^{†}\right) \end{array}\right]\left[\begin{array}{c} s_{0}I_{n}-A\\ C_{i} \end{array}\right]\right)+q+g\\ \; & =\mathrm{rank}\left[\begin{array}{c} s_{0}I_{n}-A\\ {\tilde{C}}_{i} \end{array}\right]+q+g \end{array}$$

which gives $\mathrm{rank}\left[\begin{array}{c} s_{0}I_{n}-A\\ {\tilde{C}}_{i} \end{array}\right]<n$ holds for complex $s_{0}$ with Re(s0)>0. As a result, the pair $\left(A,{\tilde{C}}_{i}\right)$ is undetectable.    □

**Lemma** **4.** *Under Assumption 2, $\mathrm{rank}\left({\tilde{C}}_{i}D\right)=q$.*

**Proof.**  $$\begin{array}{cc} \; & n+q+g_{i}=\mathrm{rank}\left[\begin{array}{ccc} I_{n} & D & 0\\ C_{i} & 0 & W_{i} \end{array}\right]\\ = & \mathrm{rank}\left(\left[\begin{array}{cc} I_{n} & 0\\ 0 & W_{i}{W_{i}}^{†}\\ 0 & I_{p_{i}}-W_{i}{W_{i}}^{†} \end{array}\right]\left[\begin{array}{ccc} I_{n} & D & 0\\ C_{i} & 0 & W_{i} \end{array}\right]\right)\\ = & \mathrm{rank}\left[\begin{array}{ccc} I_{n} & D & 0\\ W_{i}W_{i}^{†}C_{i} & 0 & W_{i}\\ C_{a,i} & 0 & 0 \end{array}\right]\\ = & \mathrm{rank}\left(\left[\begin{array}{ccc} I_{n} & D & 0\\ W_{i}W_{i}^{†}C_{i} & 0 & W_{i}\\ C_{a,i} & 0 & 0 \end{array}\right]\left[\begin{array}{ccc} I_{n} & 0 & 0\\ 0 & I_{q} & 0\\ -{W_{i}}^{†}C_{i} & 0 & I_{p_{i}} \end{array}\right]\right)\\ = & \mathrm{rank}\left[\begin{array}{ccc} I_{n} & D & 0\\ 0 & 0 & W_{i}\\ C_{a,i} & 0 & 0 \end{array}\right]=g_{i}+\mathrm{rank}\left[\begin{array}{cc} I_{n} & D\\ C_{a,i} & 0 \end{array}\right]\\ = & g_{i}+\mathrm{rank}\left(\left[\begin{array}{cc} I_{n} & 0\\ -C_{a,i} & I_{p} \end{array}\right]\left[\begin{array}{cc} I_{n} & D\\ C_{a,i} & 0 \end{array}\right]\left[\begin{array}{cc} I_{n} & -D\\ 0 & I_{p} \end{array}\right]\right)\\ = & g_{i}+\mathrm{rank}\left[\begin{array}{cc} I_{n} & 0\\ 0 & -C_{a,i}D \end{array}\right]\\ = & n+g_{i}+\mathrm{rank}\left(C_{a,i}D\right) \end{array}$$

which gives $\mathrm{rank}\left(C_{a,i}D\right)=q$. Recall that $\left[\begin{array}{c} {\tilde{C}}_{i}\\ 0_{(p_{i}-{\tilde{p}}_{i})\times n} \end{array}\right]=\Upsilon_{i}C_{a,i}$; then, we have $q=\mathrm{rank}\left(C_{a,i}D\right)=\mathrm{rank}\left(\Upsilon_{i}C_{a,i}D\right)=\mathrm{rank}\left[\begin{array}{c} {\tilde{C}}_{i}D\\ 0_{(p_{i}-{\tilde{p}}_{i})\times n} \end{array}\right]=\mathrm{rank}\left({\tilde{C}}_{i}D\right)$, which is just what we want to prove.    □

**Lemma** **5** ([36]). *$\mathrm{rank}\left({\tilde{C}}_{i}D\right)=\mathrm{rank}\left(D\right)$ if and only if there are two nonsingular matrices $T_{i}\in R^{n\times n}$ and $S_{i}\in R^{{\tilde{p}}_{i}\times {\tilde{p}}_{i}}$ such that*

(5) $$\begin{array}{c} T_{i}^{-1}D=\left[\begin{array}{c} {\bar{D}}_{i,1}\\ 0 \end{array}\right],S_{i}^{-1}{\tilde{C}}_{i}T=\left[\begin{array}{cc} {\bar{C}}_{i,1} & \\ & {\bar{C}}_{i,2} \end{array}\right] \end{array}$$

*where ${\bar{D}}_{i,1}\in R^{q\times q}$ and ${\bar{C}}_{i,1}\in R^{q\times q}$, and they are both invertible. The following Algorithm 1 will give the construction process of $T_{i}$ and $S_{i}$.*

**Algorithm 1:** Construction of transformation matrices $T_{i}$ and $S_{i}$ via singular value decomposition (SVD)

**Require:**

    Matrix ${\tilde{C}}_{i}\in R^{{\tilde{p}}_{i}\times n}$ (full row rank)
    Matrix $D\in R^{n\times q}$ (full column rank)
    Assumption: $\mathrm{rank}\left({\tilde{C}}_{i}D\right)=\mathrm{rank}\left(D\right)=q$
**Ensure:** 
    Nonsingular transformation matrix $T_{i}\in R^{n\times n}$
    Nonsingular transformation matrix $S_{i}\in R^{{\tilde{p}}_{i}\times {\tilde{p}}_{i}}$
    **Step 1:** Compute the SVD of the product matrix ${\tilde{C}}_{i}D$
    ${\tilde{C}}_{i}D=U_{{\tilde{C}}_{i}D}\Lambda_{{\tilde{C}}_{i}D}V_{{\tilde{C}}_{i}D}^{T}$
    Set $S_{i}=U_{{\tilde{C}}_{i}D}$
    **Step 2:** Compute the SVD of matrix *D*
    $D=U_{D}\Lambda_{D}V_{D}^{T}$
    **Step 3:** Compute the SVD of matrix $S_{i}^{T}{\tilde{C}}_{i}$
    $S_{i}^{T}{\tilde{C}}_{i}=U_{i}\Lambda_{i}V_{i}^{T}$
    Partition $V_{i}=\left[\begin{array}{cc} V_{i_{1}} & V_{i_{2}} \end{array}\right]$ with $V_{i_{1}}\in R^{n\times q}$, $V_{i_{2}}\in R^{n\times (n-q)}$
    **Step 4:** Construct the transformation matrix $T_{i}$
    $T_{i}=\left[\begin{array}{cc} U_{D} & V_{i_{2}} \end{array}\right]$
    **Step 5 (Optional):** Verify the canonical form
    Compute $T_{i}^{-1}D=\left[\begin{array}{c} {\bar{D}}_{i,1}\\ 0 \end{array}\right]$
    Compute $S_{i}^{-1}{\tilde{C}}_{i}T_{i}=\left[\begin{array}{cc} {\bar{C}}_{i,1} & 0\\ 0 & {\bar{C}}_{i,2} \end{array}\right]$.

**Remark** **2.** *Lemma 5 is called reduced-order technology, which is the core of dealing with UI.*

Due to Lemma 5, we have ${\bar{A}}_{i}=T_{i}^{-1}AT_{i}=\left[\begin{array}{cc} {\bar{A}}_{i,11} & {\bar{A}}_{i,12}\\ {\bar{A}}_{i,21} & {\bar{A}}_{i,22} \end{array}\right],{\bar{B}}_{i}=T_{i}^{-1}B=\left[\begin{array}{c} {\bar{B}}_{i,1}\\ {\bar{B}}_{i,2} \end{array}\right],{\bar{D}}_{i}=T_{i}^{-1}D=\left[\begin{array}{c} {\bar{D}}_{i,1}\\ 0 \end{array}\right],{\bar{C}}_{i}=S_{i}^{-1}{\tilde{C}}_{i}T_{i}=\left[\begin{array}{cc} {\bar{C}}_{i,1} & \\ & {\bar{C}}_{i,2} \end{array}\right]$. Here, ${\bar{A}}_{i,11}\in R^{q\times q}$, ${\bar{D}}_{i,1}\in R^{q\times q}$, ${\bar{C}}_{i,1}\in R^{q\times q}$ and ${\bar{C}}_{i,2}\in R^{\left({\tilde{p}}_{i}-q\right)\times \left(n-q\right)}$. Moreover, ${\bar{C}}_{i,1}$ is invariable. Now, based on Lemma 5, if we make state and output transformation of $\xi_{i}=T_{i}^{-1}x$ and $\eta_{i}=S_{i}^{-1}{\tilde{y}}_{i}$, then we have

(6) $$\begin{array}{c} \left\{\begin{array}{c} {\dot{\xi }}_{i,1}={\bar{A}}_{i,11}\xi_{i,1}+{\bar{A}}_{i,12}\xi_{i,2}+{\bar{B}}_{i,1}u+{\bar{D}}_{i,1}f\\ {\dot{\xi }}_{i,2}={\bar{A}}_{i,22}\xi_{i,2}+{\bar{A}}_{i,21}\xi_{i,1}+{\bar{B}}_{i,2}u\\ \eta_{i,1}={\bar{C}}_{i,1}\xi_{i,1}\\ \eta_{i,2}={\bar{C}}_{i,2}\xi_{i,2} \end{array}\right. \end{array}$$

where $\xi_{i}={\left[\begin{array}{cc} \xi_{i,1}^{T} & \xi_{i,2}^{T} \end{array}\right]}^{T}$ and $\eta_{i}={\left[\begin{array}{cc} \eta_{i,1}^{T} & \eta_{i,2}^{T} \end{array}\right]}^{T}$ with $\xi_{i,1}\in R^{q},\xi_{i,2}\in R^{n-q},\eta_{i,1}\in R^{q}$ and $\eta_{i,2}\in R^{{\tilde{p}}_{i}-q}$.

**Lemma** **6.** *The pair $\left({\bar{A}}_{i,22},{\bar{C}}_{i,2}\right)$ is undetectable.*

**Proof.**  Since the pair $\left(A,{\tilde{C}}_{i}\right)$ is undetectable, derived from Lemma 3, there exists at least a complex $s_{i,0}$ with Re(si,0) such that

$$\begin{array}{cc} \; & n+q<\mathrm{rank}\left[\begin{array}{cc} s_{i,0}I_{n}-A & D\\ {\tilde{C}}_{i} & 0 \end{array}\right]\\ & =\mathrm{rank}\left(\left[\begin{array}{cc} T_{i}^{-1} & 0\\ 0 & S_{i}^{-1} \end{array}\right]\left[\begin{array}{cc} s_{i,0}I_{n}-A & D\\ {\tilde{C}}_{i} & 0 \end{array}\right]\left[\begin{array}{cc} T_{i} & 0\\ 0 & I_{q} \end{array}\right]\right)\\ \; & =\mathrm{rank}\left[\begin{array}{cc} s_{i,0}I_{n}-{\bar{A}}_{i} & {\bar{D}}_{i}\\ {\bar{C}}_{i} & 0 \end{array}\right]\\ \; & =\mathrm{rank}\left[\begin{array}{ccc} s_{i,0}I_{q}-{\bar{A}}_{i,11} & -{\bar{A}}_{i,11} & {\bar{D}}_{i,1}\\ -{\bar{A}}_{i,12} & s_{i,0}I_{n-q}-{\bar{A}}_{i,22} & 0\\ {\bar{C}}_{i,1} & 0 & 0\\ 0 & {\bar{C}}_{i,2} & 0 \end{array}\right]. \end{array}$$

Noticing that ${\bar{D}}_{i,1}$ and ${\bar{C}}_{i,1}$ are both invertible, thus

$$\begin{array}{cc} \; & n+q<\mathrm{rank}\left[\begin{array}{ccc} s_{i,0}I_{q}-{\bar{A}}_{i,11} & -{\bar{A}}_{i,11} & {\bar{D}}_{i,1}\\ -{\bar{A}}_{i,12} & s_{i,0}I_{n-q}-{\bar{A}}_{i,22} & 0\\ {\bar{C}}_{i,1} & 0 & 0\\ 0 & {\bar{C}}_{i,2} & 0 \end{array}\right]\\ \; & =\mathrm{rank}\left[\begin{array}{c} s_{i,0}I_{n-q}-{\bar{A}}_{i,22}\\ {\bar{C}}_{i,2} \end{array}\right]+q+q \end{array}$$

So, we can conclude that the pair $\left({\bar{A}}_{i,22},{\bar{C}}_{i,2}\right)$ is undetectable, based on $\mathrm{rank}\left[\begin{array}{c} s_{i,0}I_{n-q}-{\bar{A}}_{i,22}\\ {\bar{C}}_{i,2} \end{array}\right]<n-q$. □

Since the pair $\left({\bar{A}}_{i,22},{\bar{C}}_{i,2}\right)$ is undetectable, we can construct an orthogonal matrix $\Omega_{i}\in R^{\left(n-q\right)\times \left(n-q\right)}$ such that $\Omega_{i}{\bar{A}}_{i,22}\Omega_{i}^{T}=\left[\begin{array}{cc} A_{id} & 0\\ A_{ir} & A_{iu} \end{array}\right]$ and ${\bar{C}}_{i,2}\Omega_{i}^{T}=\left[\begin{array}{cc} C_{id} & 0 \end{array}\right]$, and moreover, the pair $\left(A_{id},C_{id}\right)$ is detectable. Here, $A_{id}\in R^{\left(n-q-r_{i}\right)\times \left(n-q-r_{i}\right)},A_{ir}\in R^{r_{i}\times \left(n-q-r_{i}\right)}$ and $A_{iu}\in R^{r_{i}\times r_{i}}$ with $r_{i}$ being the number of undetectable states of $\left({\bar{A}}_{i,22},{\bar{C}}_{i,2}\right)$.

Now, make a state transformation $z_{i}=\Omega_{i}\xi_{i,2}$, and the second and the third equations of (6) become

(7) $$\begin{array}{c} \left\{\begin{array}{c} {\dot{z}}_{i}=\Omega_{i}{\bar{A}}_{i,22}\Omega_{i}^{T}z_{i}+\Omega_{i}{\bar{A}}_{i,21}{\bar{C}}_{i,1}^{-1}\eta_{i,1}+\Omega_{i}{\bar{B}}_{i,2}u\\ \eta_{i,2}={\bar{C}}_{i,2}\Omega_{i}^{T}z_{i} \end{array}\right. \end{array}$$

Partition $\Omega_{i}$ into $\Omega_{i}={\left[\begin{array}{cc} \Omega_{i,1}^{T} & \Omega_{i,2}^{T} \end{array}\right]}^{T}$, where $\Omega_{i,1}\in R^{\left(n-q-r_{i}\right)\times \left(n-q\right)}$ and $\Omega_{i,2}\in R^{r_{i}\times \left(n-q\right)}$. Next, consider the following distributed observer for system (7).

(8) $$\begin{array}{cc} {\dot{\hat{z}}}_{i}= & \Omega_{i}{\bar{A}}_{i,22}\Omega_{i}^{T}{\hat{z}}_{i}+\Omega_{i}{\bar{A}}_{i,21}{\bar{C}}_{i,1}^{-1}\eta_{i,1}+\Omega_{i}{\bar{B}}_{i,2}u+\Omega_{i}K_{i}\left(\eta_{i,2}-{\bar{C}}_{i,2}\Omega_{i}^{T}{\hat{z}}_{i}\right)\\ & -cH_{i}V_{i_{2}}^{T}\sum_{j=1}^{N}l_{ij}\left(T_{i}\left[\begin{array}{c} {\bar{C}}_{i,1}^{-1}\eta_{i,1}\\ \Omega_{i}^{T}{\hat{z}}_{i} \end{array}\right]-T_{j}\left[\begin{array}{c} {\bar{C}}_{j,1}^{-1}\eta_{j,1}\\ \Omega_{j}^{T}{\hat{z}}_{j} \end{array}\right]\right) \end{array}$$

where $K_{i}\in R^{\left(n-q\right)\times \left({\tilde{p}}_{i}-q\right)}$, and c>0 are the observer gains to be designed later, and $H_{i}=\Omega_{i}\Omega_{i,2}^{T}\Omega_{i,2}=\left[\begin{array}{c} 0_{\left(n-q-r_{i}\right)\times \left(n-q\right)}\\ \Omega_{i,2} \end{array}\right]$.

Combining (7) and (8), as well as denoting ${\tilde{z}}_{i}=z_{i}-{\hat{z}}_{i}$, yields the observer error dynamics:

$$\begin{array}{cc} {\dot{\tilde{z}}}_{i} & =\Omega_{i}{\bar{A}}_{i,22}\Omega_{i}^{T}{\tilde{z}}_{i}-\Omega_{i}K_{i}\left(\eta_{i,2}-{\bar{C}}_{i,2}\Omega_{i}^{T}{\hat{z}}_{i}\right)+cH_{i}V_{i_{2}}^{T}\sum_{j=1}^{N}l_{ij}\left(T_{i}\left[\begin{array}{c} {\bar{C}}_{i,1}^{-1}\eta_{i,1}\\ \Omega_{i}^{T}{\hat{z}}_{i} \end{array}\right]-T_{j}\left[\begin{array}{c} {\bar{C}}_{j,1}^{-1}\eta_{j,1}\\ \Omega_{j}^{T}{\hat{z}}_{j} \end{array}\right]\right)\\ \; & =\Omega_{i}{\bar{A}}_{i,22}\Omega_{i}^{T}{\tilde{z}}_{i}-\Omega_{i}K_{i}\left({\bar{C}}_{i,2}\Omega_{i}^{T}z_{i}-{\bar{C}}_{i,2}\Omega_{i}^{T}{\hat{z}}_{i}\right)\\ & \;\;\;\;\;\;+cH_{i}V_{i_{2}}^{T}\sum_{j=1}^{N}l_{ij}\left(T_{i}\left[\begin{array}{c} {\bar{C}}_{i,1}^{-1}\eta_{i,1}\\ \Omega_{i}^{T}{\hat{z}}_{i} \end{array}\right]-T_{j}\left[\begin{array}{c} {\bar{C}}_{j,1}^{-1}\eta_{j,1}\\ \Omega_{j}^{T}{\hat{z}}_{j} \end{array}\right]\right)\\ \; & =\Omega_{i}{\bar{A}}_{i,22}\Omega_{i}^{T}{\tilde{z}}_{i}-\Omega_{i}K_{i}{\bar{C}}_{i,2}\Omega_{i}^{T}{\tilde{z}}_{i}+cH_{i}V_{i_{2}}^{T}\sum_{j=1}^{N}l_{ij}\left(T_{i}\left[\begin{array}{c} {\bar{C}}_{i,1}^{-1}\eta_{i,1}\\ \Omega_{i}^{T}{\hat{z}}_{i} \end{array}\right]-T_{j}\left[\begin{array}{c} {\bar{C}}_{j,1}^{-1}\eta_{j,1}\\ \Omega_{j}^{T}{\hat{z}}_{j} \end{array}\right]\right)\\ \; & =\left(\Omega_{i}{\bar{A}}_{i,22}\Omega_{i}^{T}-\Omega_{i}K_{i}{\bar{C}}_{i,2}\Omega_{i}^{T}\right){\tilde{z}}_{i}+cH_{i}V_{i_{2}}^{T}\sum_{j=1}^{N}l_{ij}\left(T_{i}\left[\begin{array}{c} {\bar{C}}_{i,1}^{-1}\eta_{i,1}\\ \Omega_{i}^{T}{\hat{z}}_{i} \end{array}\right]-T_{j}\left[\begin{array}{c} {\bar{C}}_{j,1}^{-1}\eta_{j,1}\\ \Omega_{j}^{T}{\hat{z}}_{j} \end{array}\right]\right). \end{array}$$

If $K_{i}=\Omega_{i}^{T}\left[\begin{array}{c} K_{id}\\ 0_{r_{i}\times \left({\tilde{p}}_{i}-q\right)} \end{array}\right]$ with $K_{id}\in R^{\left(n-q-r_{i}\right)\times \left({\tilde{p}}_{i}-q\right)}$, then the above equation becomes

(9) $$\begin{array}{c} {\dot{\tilde{z}}}_{i}={\overset{⌣}{A}}_{i}{\tilde{z}}_{i}+cH_{i}V_{i_{2}}^{T}\sum_{j=1}^{N}l_{ij}\left(T_{i}\left[\begin{array}{c} {\bar{C}}_{i,1}^{-1}\eta_{i,1}\\ \Omega_{i}^{T}{\hat{z}}_{i} \end{array}\right]-T_{j}\left[\begin{array}{c} {\bar{C}}_{j,1}^{-1}\eta_{j,1}\\ \Omega_{j}^{T}{\hat{z}}_{j} \end{array}\right]\right) \end{array}$$

where ${\overset{⌣}{A}}_{i}=\left[\begin{array}{cc} A_{id}-K_{id}C_{id} & 0\\ A_{ir} & A_{iu} \end{array}\right]$. Moreover

$$\begin{array}{cc} \; & cH_{i}V_{i_{2}}^{T}\sum_{j=1}^{N}l_{ij}\left(T_{i}\left[\begin{array}{c} {\bar{C}}_{i,1}^{-1}\eta_{i,1}\\ \Omega_{i}^{T}{\hat{z}}_{i} \end{array}\right]-T_{j}\left[\begin{array}{c} {\bar{C}}_{j,1}^{-1}\eta_{j,1}\\ \Omega_{j}^{T}{\hat{z}}_{j} \end{array}\right]\right)\\ \; & =cH_{i}V_{i_{2}}^{T}\sum_{j=1}^{N}l_{ij}\left(T_{i}\left[\begin{array}{c} {\bar{C}}_{i,1}^{-1}\eta_{i,1}\\ \Omega_{i}^{T}{\hat{z}}_{i} \end{array}\right]-x+x-T_{j}\left[\begin{array}{c} {\bar{C}}_{j,1}^{-1}\eta_{j,1}\\ \Omega_{j}^{T}{\hat{z}}_{j} \end{array}\right]\right)\\ \; & =cH_{i}V_{i_{2}}^{T}\sum_{j=1}^{N}l_{ij}\left(T_{i}\left[\begin{array}{c} {\bar{C}}_{i,1}^{-1}\eta_{i,1}\\ \Omega_{i}^{T}{\hat{z}}_{i} \end{array}\right]-T_{i}\left[\begin{array}{c} {\bar{C}}_{i,1}^{-1}\eta_{i,1}\\ \Omega_{i}^{T}z_{i} \end{array}\right]+T_{j}\left[\begin{array}{c} {\bar{C}}_{j,1}^{-1}\eta_{j,1}\\ \Omega_{j}^{T}z_{j} \end{array}\right]-T_{j}\left[\begin{array}{c} {\bar{C}}_{j,1}^{-1}\eta_{j,1}\\ \Omega_{j}^{T}{\hat{z}}_{j} \end{array}\right]\right)\\ \; & =-cH_{i}V_{i_{2}}^{T}\sum_{j=1}^{N}l_{ij}\left(T_{i}\left[\begin{array}{c} 0\\ \Omega_{i}^{T}{\tilde{z}}_{i} \end{array}\right]-T_{j}\left[\begin{array}{c} 0\\ \Omega_{j}^{T}{\tilde{z}}_{j} \end{array}\right]\right)\\ \; & =-cH_{i}V_{i_{2}}^{T}\sum_{j=1}^{N}l_{ij}\left(V_{i_{2}}\Omega_{i}^{T}{\tilde{z}}_{i}-V_{j_{2}}\Omega_{j}^{T}{\tilde{z}}_{j}\right). \end{array}$$

That is, we have

(10) $$\begin{array}{c} {\dot{\tilde{z}}}_{i}={\overset{⌣}{A}}_{i}{\tilde{z}}_{i}-cH_{i}V_{i_{2}}^{T}\sum_{j=1}^{N}l_{ij}\left(V_{i_{2}}\Omega_{i}^{T}{\tilde{z}}_{i}-V_{j_{2}}\Omega_{j}^{T}{\tilde{z}}_{j}\right) \end{array}$$

Denote ${\tilde{z}}_{i}=\left[\begin{array}{c} {\tilde{z}}_{id}\\ {\tilde{z}}_{iu} \end{array}\right]$, where ${\tilde{z}}_{id}\in R^{\left(n-q-r_{i}\right)}$, and ${\tilde{z}}_{iu}\in R^{r_{i}}$, then (9) can be rewritten as

(11) $$\begin{array}{c} \left\{\begin{array}{c} {\dot{\tilde{z}}}_{id}=A_{id}-K_{id}C_{id}{\tilde{z}}_{id}\\ {\dot{\tilde{z}}}_{iu}=A_{ir}{\tilde{z}}_{id}+A_{iu}{\tilde{z}}_{iu}c\Omega_{i,2}V_{i_{2}}^{T}\sum_{j=1}^{N}l_{ij}\left(V_{i_{2}}\Omega_{i,1}^{T}{\tilde{z}}_{id}-V_{i_{2}}\Omega_{j,1}^{T}{\tilde{z}}_{jd}\right)\\ \;\;\;\;\;\;\;\;\;-c\Omega_{i,2}V_{i_{2}}^{T}\sum_{j=1}^{N}l_{ij}\left(V_{i_{2}}\Omega_{i,2}^{T}{\tilde{z}}_{iu}-V_{i_{2}}\Omega_{j,2}^{T}{\tilde{z}}_{ju}\right) \end{array}\right. \end{array}$$

The overall system of (11) is

(12) $$\begin{array}{c} \left[\begin{array}{c} {\dot{\tilde{z}}}_{d}\\ {\dot{\tilde{z}}}_{u} \end{array}\right]=\left[\begin{array}{cc} A_{d}-K_{d}C_{d} & 0\\ A_{r}-c\Omega_{2}V_{2}^{T}\left(L⊗I_{n}\right)V_{2}\Omega_{1}^{T} & A_{u}-c\Omega_{2}V_{2}^{T}\left(L⊗I_{n}\right)V_{2}\Omega_{2}^{T} \end{array}\right]\left[\begin{array}{c} {\tilde{z}}_{d}\\ {\tilde{z}}_{u} \end{array}\right] \end{array}$$

where $A_{d}={\mathrm{diag}}_{i\in N}\left(A_{id}\right),K_{d}={\mathrm{diag}}_{i\in N}\left(K_{id}\right),C_{d}={\mathrm{diag}}_{i\in N}\left(C_{id}\right),V_{2}={\mathrm{diag}}_{i\in N}\left(V_{i_{2}}\right),\Omega_{1}={\mathrm{diag}}_{i\in N}\left(\Omega_{i,1}\right),\Omega_{2}={\mathrm{diag}}_{i\in N}\left(\Omega_{i,2}\right),{\tilde{z}}_{d}={\left[\begin{array}{ccc} {\tilde{z}}_{1d}^{T} & \cdots & {\tilde{z}}_{Nd}^{T} \end{array}\right]}^{T}$ and ${\tilde{z}}_{u}={\left[\begin{array}{ccc} {\tilde{z}}_{1u}^{T} & \cdots & {\tilde{z}}_{Nu}^{T} \end{array}\right]}^{T}$.

**Lemma** **7** ([23]). *Under Assumption A1, if we choose*

(13) $$\begin{array}{c} c>\frac{2∥A_{u}∥}{\lambda_{\min}\left(\Omega_{2}V_{2}^{T}\left(\hat{L}⊗I_{n}\right)V_{2}\Omega_{2}^{T}\right)}+\delta_{0} \end{array}$$

*for some constant $\delta_{0}>0$, then matrix $A_{u}-c\Omega_{2}V_{2}^{T}\left(L⊗I_{n}\right)V_{2}\Omega_{2}^{T}$ is a Hurwitz matrix.*

Now, we can present the main result of this paper using the following theorem.

**Theorem** **1.** *With Assumptions 1 and 2, if the topology is a strong connected, then (12) is a reduced-order DUIO for the multiple sensor system (1) such that $\lim_{t\to \infty }{\tilde{z}}_{i}\left(t\right)=0$ and thus $\lim_{t\to \infty }{\tilde{x}}_{i}\left(t\right)=0$, where ${\tilde{x}}_{i}=x-{\hat{x}}_{i}$ with ${\hat{x}}_{i}=T_{i}\left[\begin{array}{c} {\bar{C}}_{i,1}^{-1}\eta_{i,1}\\ \Omega_{i}^{-1}{\hat{z}}_{i} \end{array}\right]$.*

**Proof.**  We only need to prove that ${\left[\begin{array}{cc} {{\tilde{z}}_{id}}^{T} & {{\tilde{z}}_{iu}}^{T} \end{array}\right]}^{T}\to 0$. On the one hand, since the pair $\left(A_{d},C_{d}\right)$ is detectable, we can determine the matrix $K_{d}$ such that the all eigenvalues of the resulting matrix $A_{d}-K_{d}C_{d}$ are with negative real parts. On the other hand, Lemma 7 guarantees that if we choose a *c* value that is large enough such that (13) holds, then the matrix $A_{u}-c\Omega_{2}V_{2}^{T}\left(L⊗I_{n}\right)V_{2}\Omega_{2}^{T}$ is a Hurwitz matrix. Therefore, the matrix in (12) is a Hurwitz matrix, which means that dynamic system (12) is asymptotically stable. Consequently, we have ${\left[\begin{array}{cc} {{\tilde{z}}_{id}}^{T} & {{\tilde{z}}_{iu}}^{T} \end{array}\right]}^{T}\to 0$, i.e., $\lim_{t\to \infty }{\tilde{z}}_{i}\left(t\right)=0$. Since ${\tilde{x}}_{i}=x-{\hat{x}}_{i}=T_{i}\left[\begin{array}{c} 0\\ \Omega_{i}^{-1}{\tilde{z}}_{i} \end{array}\right]$, we have $\lim_{t\to \infty }{\tilde{x}}_{i}\left(t\right)=0$. □

**Remark** **3.** *In computational complexity, all complex matrix operations, including the derivation of transformation matrices (e.g., $T_{i},S_{i},\Omega_{i}$) and the calculation of observer gains (e.g., $K_{i},c$), are performed offline. These are one-time computations and do not contribute to the online burden. And thanks for the reduced order observer, the computational complexity of observer gain $\left(n-q\right)\times \left({\tilde{p}}_{i}-q\right)$ reduces to $\left(n-q-r_{i}\right)\times \left({\tilde{p}}_{i}-q\right)$.*

### 3.2. UIR via Interval Observer

In this subsection, a local interval observer is designed in advance and an correlation between the UI and the state is set up. In this way, a UIR method is developed, and the UIR is able to estimate the actual UI asymptotically.

According to the first Equation (6), we can deduce that

(14) $$\begin{array}{c} {\dot{\eta }}_{i,1}={\tilde{A}}_{i,11}\eta_{i,1}+{\bar{C}}_{i,1}{\bar{A}}_{i,12}\xi_{i,2}+{\bar{C}}_{i,1}{\bar{B}}_{i,1}u+{\bar{C}}_{i,1}{\bar{D}}_{i,1}f \end{array}$$

where ${\tilde{A}}_{i,11}={\bar{C}}_{i,1}{\bar{A}}_{i,11}{\bar{C}}_{i,1}^{-1}$. For (14), we design an interval observer as follows

(15) $$\begin{array}{c} \left\{\begin{array}{c} {\dot{\bar{\eta }}}_{i,1}={\tilde{A}}_{i,11}\eta_{i,1}+{\bar{C}}_{i,1}{\bar{A}}_{i,12}{\hat{\xi }}_{i,2}+{\bar{C}}_{i,1}{\bar{B}}_{i,1}u+M\left({\bar{\eta }}_{i,1}-\eta_{i,1}\right)+{\left({\bar{C}}_{i,1}{\bar{D}}_{i,1}\right)}^{+}\bar{f}-{\left({\bar{C}}_{i,1}{\bar{D}}_{i,1}\right)}^{-}\underline{f}\\ {\dot{\underline{\eta }}}_{i,1}={\tilde{A}}_{i,11}\eta_{i,1}+{\bar{C}}_{i,1}{\bar{A}}_{i,12}{\hat{\xi }}_{i,2}+{\bar{C}}_{i,1}{\bar{B}}_{i,1}u+M\left({\underline{\eta }}_{i,1}-\eta_{i,1}\right)+{\left({\bar{C}}_{i,1}{\bar{D}}_{i,1}\right)}^{+}\underline{f}-{\left({\bar{C}}_{i,1}{\bar{D}}_{i,1}\right)}^{-}\bar{f} \end{array}\right. \end{array}$$

where ${\hat{\xi }}_{i,2}$ is produced by the reduced-order observer (11). Moreover, (15) gives

(16) $$\begin{array}{c} {\dot{\overset{˘}{\eta }}}_{i,1}=M{\tilde{\eta }}_{i,1}+\left|{\bar{C}}_{i,1}{\bar{D}}_{i,1}\right|\overset{˘}{f} \end{array}$$

where ${\overset{˘}{\eta }}_{i,1}={\bar{\eta }}_{i,1}-{\underline{\eta }}_{i,1}$ and $\overset{˘}{f}=\bar{f}-\underline{f}$.

**Lemma** **8** ([41]). *Let x(t) satisfy $\underline{x}\left(t\right)⩽x\left(t\right)⩽\bar{x}\left(t\right)$. Then, for any constant matrix $X\in R^{m\times n}$, $X^{+}\underline{x}\left(t\right)-X^{-}\bar{x}\left(t\right)⩽Xx\left(t\right)⩽X^{+}\bar{x}\left(t\right)-X^{-}\underline{x}\left(t\right)$.*

**Lemma** **9** ([41]). *Suppose that R is Metzler, ς(t)⩾0, and x(0)⩾0 in the system $\dot{x}=Rx+\varsigma \left(t\right)$, then x(t)⩾0 for all t≥0.*

**Theorem** **2.** *Under Assumptions 1 and 2, system (15) is an interval observer of (14) such that ${\underline{\eta }}_{i,1}\leq \eta_{i,1}\leq {\bar{\eta }}_{i,1}$ holds for all $t>c_{0}$ with scalar $c_{0}>0$, If a Metzler and Hurwitz matrix M is arbitrarily selected and the initial values are assigned ${\bar{\eta }}_{i,1}\left(0\right)={\tilde{\bar{C}}}_{i,1}^{+}{\bar{x}}_{0}-{\tilde{\bar{C}}}_{i,1}^{-}{\underline{x}}_{0}$ and ${\underline{\eta }}_{i,1}\left(0\right)={\tilde{\bar{C}}}_{i,1}^{+}{\underline{x}}_{0}-{\tilde{\bar{C}}}_{i,1}^{-}{\bar{x}}_{0}$, where ${\tilde{\bar{C}}}_{i,1}={\bar{C}}_{i,1}\left[\begin{array}{cc} I_{q} & 0 \end{array}\right]T^{-1}$.*

**Proof.**  Using (14) and (15), we obtain the interval observer error dynamics as

(17) $$\begin{array}{c} \left\{\begin{array}{c} {\dot{\bar{e}}}_{\eta_{i,1}}=M{\bar{e}}_{\eta_{i,1}}-{\bar{C}}_{i,1}{\bar{A}}_{i,12}{\tilde{\xi }}_{i,2}-{\bar{C}}_{i,1}{\bar{D}}_{i,1}f+{\left({\bar{C}}_{i,1}{\bar{D}}_{i,1}\right)}^{+}\bar{f}-{\left({\bar{C}}_{i,1}{\bar{D}}_{i,1}\right)}^{-}\underline{f}\\ {\dot{\underline{e}}}_{\eta_{i,1}}=M{\underline{e}}_{\eta_{i,1}}+{\bar{C}}_{i,1}{\bar{A}}_{i,12}{\tilde{\xi }}_{i,2}+{\bar{C}}_{i,1}{\bar{D}}_{i,1}f-{\left({\bar{C}}_{i,1}{\bar{D}}_{i,1}\right)}^{+}\underline{f}+{\left({\bar{C}}_{i,1}{\bar{D}}_{i,1}\right)}^{-}\bar{f} \end{array}\right. \end{array}$$

where ${\bar{e}}_{\eta_{i,1}}={\bar{\eta }}_{i,1}-\eta_{i,1}$ and ${\underline{e}}_{\eta_{i,1}}=\eta_{i,1}-{\underline{\eta }}_{i,1}$. By Lemma 8, we can choose suitable $\bar{f}$ and $\underline{f}$ such that ${\bar{C}}_{i,1}{\bar{D}}_{i,1}f-\left({\left({\bar{C}}_{i,1}{\bar{D}}_{i,1}\right)}^{+}\underline{f}-{\left({\bar{C}}_{i,1}{\bar{D}}_{i,1}\right)}^{-}\bar{f}\right)>0$ and $\left({\left({\bar{C}}_{i,1}{\bar{D}}_{i,1}\right)}^{+}\bar{f}-{\left({\bar{C}}_{i,1}{\bar{D}}_{i,1}\right)}^{-}\underline{f}\right)-{\bar{C}}_{i,1}{\bar{D}}_{i,1}f>0$ hold. Since $\lim_{t\to \infty }{\tilde{z}}_{i}\left(t\right)=0$. Therefore, there exists $c_{0}>0$ such that ${\bar{C}}_{i,1}{\bar{A}}_{i,12}{\tilde{\xi }}_{i,2}+{\bar{C}}_{i,1}{\bar{D}}_{i,1}f-\left({\left({\bar{C}}_{i,1}{\bar{D}}_{i,1}\right)}^{+}\underline{f}-{\left({\bar{C}}_{i,1}{\bar{D}}_{i,1}\right)}^{-}\bar{f}\right)>0$ and $-{\bar{C}}_{i,1}{\bar{A}}_{i,12}{\tilde{\xi }}_{i,2}-{\bar{C}}_{i,1}{\bar{D}}_{i,1}f+\left({\left({\bar{C}}_{i,1}{\bar{D}}_{i,1}\right)}^{+}\bar{f}-{\left({\bar{C}}_{i,1}{\bar{D}}_{i,1}\right)}^{-}\underline{f}\right)>0$ hold for all $t\geq c_{0}$. Additionally, since $\eta_{i,1}={\tilde{\bar{C}}}_{i,1}x$, then, under Assumption 2, and by Lemma 8, one has ${\underline{\eta }}_{i,1}\left(0\right)\leq \eta_{i,1}\left(0\right)\leq {\bar{\eta }}_{i,1}\left(0\right)$ which implies that ${\bar{e}}_{\eta_{i,1}}\geq 0$ and ${\underline{e}}_{\eta_{i,1}}\geq 0$. Under the condition that matrix *M* is both Hurwitz and Metzler, Lemma 9 directly yields ${\underline{\eta }}_{i,1}\left(t\right)\leq \eta_{i,1}\left(t\right)\leq {\bar{\eta }}_{i,1}\left(t\right)$ for all t≥0. □

To build up a relationship between the UI and the state estimation error from the output bounds algebraically, we first note that ${\underline{\eta }}_{i,1}\leq \eta_{i,1}\leq {\bar{\eta }}_{i,1}$. This inequality guarantees the existence of a time-varying variable $\theta_{i}\left(t\right)\in R^{q}$ such that

(18) $$\begin{array}{c} \eta_{i,1}=\mathrm{diag}\left({\overset{˘}{\eta }}_{i,1}\right)\theta_{i}+{\underline{\eta }}_{i,1} \end{array}$$

where $\theta_{i}={\left[\begin{array}{ccc} \theta_{i1} & \cdots & \theta_{iq} \end{array}\right]}^{T}$ with $0\leq \theta_{ij}\leq 1\left(j=1,\cdots ,q\right)$. Then, one has

(19) $$\begin{array}{c} \theta_{i}=\left(\mathrm{diag}{\left({\overset{˘}{\eta }}_{i,1}+\varepsilon_{i}\right)}^{-1}-\mathrm{diag}\left(\varepsilon_{i}\right)\right)\left(\eta_{i,1}-{\underline{\eta }}_{i,1}\right) \end{array}$$

where time-varying variable $\varepsilon_{i}={\left[\begin{array}{ccc} \varepsilon_{i1} & \cdots & \varepsilon_{iq} \end{array}\right]}^{T}$ with $\varepsilon_{ij}=1$ if ${\overset{˘}{\eta }}_{i,1j}=0$, otherwise $\varepsilon_{ij}=0$, where ${\overset{˘}{\eta }}_{i,1j}$ is the *j*th entry of ${\overset{˘}{\eta }}_{i,1}\left(j=1,\cdots ,q\right)$. Meanwhile, by (18), we have

(20) $$\begin{array}{c} {\dot{\eta }}_{i,1}=\mathrm{diag}\left({\dot{\overset{˘}{\eta }}}_{i,1}\right)\theta_{i}+\mathrm{diag}\left({\overset{˘}{\eta }}_{i,1}\right){\dot{\theta }}_{i}+{\dot{\underline{\eta }}}_{i,1} \end{array}$$

Consequently, based on (16) and (15), (20) gives

(21) $$\begin{array}{c} {\dot{\eta }}_{i,1}=\mathrm{diag}\left(\phi_{1}\right)\theta_{i}+\mathrm{diag}\left({\overset{˘}{\eta }}_{i,1}\right){\dot{\theta }}_{i}+\phi_{2} \end{array}$$

where $\phi_{1}=M{\overset{˘}{\eta }}_{i,1}+\left|{\bar{C}}_{i,1}{\bar{D}}_{1}\right|\overset{˘}{f}$ and $\phi_{2}={\tilde{A}}_{i,11}\eta_{i,1}+{\bar{C}}_{i,1}{\bar{A}}_{i,12}{\hat{\xi }}_{i,2}+{\bar{C}}_{i,1}{\bar{B}}_{i,1}u+{\left({\bar{C}}_{i,1}{\bar{D}}_{i,1}\right)}^{+}\underline{f}-{\left({\bar{C}}_{i,1}{\bar{D}}_{i,1}\right)}^{-}\bar{f}+M\left({\underline{\eta }}_{i,1}-\eta_{i,1}\right)$. Now, when we compare (14) with (21), we obtain

(22) $$\begin{array}{cc} {\bar{C}}_{i,1}{\bar{D}}_{i,1}f & =\mathrm{diag}\left(\phi_{1}\right)\theta_{i}+\mathrm{diag}\left({\overset{˘}{\eta }}_{i,1}\right){\dot{\theta }}_{i}-{\bar{C}}_{i,1}{\bar{A}}_{i,12}{\tilde{\xi }}_{i,2}\\ & +{\left({\bar{C}}_{i,1}{\bar{D}}_{i,1}\right)}^{+}\underline{f}-{\left({\bar{C}}_{i,1}{\bar{D}}_{i,1}\right)}^{-}\bar{f}+M\left({\underline{\eta }}_{i,1}-\eta_{i,1}\right) \end{array}$$

By Lemma 4, $\mathrm{rank}\left({\tilde{C}}_{i}D\right)=\mathrm{rank}\left(D\right)$. Thereby, we can deduce that

$$\begin{array}{cc} \; & \mathrm{rank}\left({\bar{C}}_{i,1}{\bar{D}}_{i,1}\right)=\mathrm{rank}\left({\tilde{C}}_{i}{\bar{D}}_{i,1}\right)\\ & =\mathrm{rank}\left(S_{i}{\tilde{C}}_{i}{\bar{D}}_{i,1}\right)=\mathrm{rank}\left(S_{i}S_{i}^{-1}{\tilde{C}}_{i}T_{i}T_{i}^{-1}D\right)\\ & =\mathrm{rank}\left({\tilde{C}}_{i}D\right)=\mathrm{rank}\left(D\right)=\mathrm{rank}\left(T_{i}^{-1}D\right)=\mathrm{rank}\left({\bar{D}}_{i,1}\right) \end{array}$$

which means that

$$\begin{array}{c} \left({\bar{C}}_{i,1}{\bar{D}}_{i,1}\right)={\left[{\left({\bar{C}}_{i,1}{\bar{D}}_{i,1}\right)}^{T}\left({\bar{C}}_{i,1}{\bar{D}}_{i,1}\right)\right]}^{-1}{\left({\bar{C}}_{i,1}{\bar{D}}_{i,1}\right)}^{T} \end{array}$$

exists. As a result, we have

(23) $$\begin{array}{cc} f= & {\left({\bar{C}}_{i,1}{\bar{D}}_{i,1}\right)}^{†}(\mathrm{diag}\left(\phi_{1}\right)\theta_{i}+\mathrm{diag}\left({\overset{˘}{\eta }}_{i,1}\right){\dot{\theta }}_{i}-{\bar{C}}_{i,1}{\bar{A}}_{i,12}{\tilde{\xi }}_{i,2}\\ & +M\left({\underline{\eta }}_{i,1}-\eta_{i,1}\right)+{\left({\bar{C}}_{i,1}{\bar{D}}_{i,1}\right)}^{+}\underline{f}-{\left({\bar{C}}_{i,1}{\bar{D}}_{i,1}\right)}^{-}\bar{f}) \end{array}$$

Now, by referring to (23), a UIR method is developed as

(24) $$\begin{array}{cc} {\hat{f}}_{i}= & {\left({\bar{C}}_{i,1}{\bar{D}}_{i,1}\right)}^{†}(\mathrm{diag}\left(\phi_{1}\right)\theta_{i}+\mathrm{diag}\left({\overset{˘}{\eta }}_{i,1}\right){\hat{\dot{\theta }}}_{i}-{\bar{C}}_{i,1}{\bar{A}}_{i,12}{\tilde{\xi }}_{i,2}\\ & +M\left({\underline{\eta }}_{i,1}-\eta_{i,1}\right)+{\left({\bar{C}}_{i,1}{\bar{D}}_{i,1}\right)}^{+}\underline{f}-{\left({\bar{C}}_{i,1}{\bar{D}}_{i,1}\right)}^{-}\bar{f}) \end{array}$$

Here, ${\hat{\dot{\theta }}}_{i}$ is the finite-time estimate of ${\dot{\theta }}_{i}$, produced via the following differentiator [42]:

(25) $$\begin{array}{c} \left\{\begin{array}{c} {\dot{\zeta }}_{1,ij}=\kappa_{1,ij}=-\rho_{1,ij}{\left|\zeta_{1,ij}-\theta_{ij}\right|}^{\frac{1}{2}}\mathrm{sign}\left(\zeta_{1,ij}-\theta_{ij}\right)+\zeta_{2,ij}\\ {\dot{\zeta }}_{2,ij}=-\rho_{2,ij}\mathrm{sign}\left(\zeta_{2,ij}-\kappa_{1,ij}\right),\left(j=1,\cdots ,q\right) \end{array}\right. \end{array}$$

where $\rho_{1,ij}$ and $\rho_{2,ij}$ denote positive tuning gains whose values require proper selection. Therefore, $\zeta_{2,ij}$ provides a finite-time estimate of ${\dot{\theta }}_{ij}$, and hence ${\hat{\dot{\theta }}}_{ij}={\left[\begin{array}{ccc} \zeta_{2,i1} & \cdots & \zeta_{2,iq} \end{array}\right]}^{T}$ consequently forms the corresponding finite-time estimate of ${\dot{\theta }}_{ij}$.

Moreover, a reconstruction for the measurement noise can be obtained by

(26) $$\begin{array}{c} \begin{array}{c} {\hat{\omega }}_{i}=W_{i}^{†}\left(y_{i}-C_{i}{\hat{x}}_{i}\right) \end{array} \end{array}$$

where ${\hat{x}}_{i}$ is provided by Theorem 1 and $W_{i}^{†}={\left(W_{i}^{T}W_{i}\right)}^{-1}W_{i}^{T}$. Obviously, we have $\lim_{t\to \infty }{\tilde{\omega }}_{i}\left(t\right)=0$, where ${\tilde{\omega }}_{i}\left(t\right)=\omega_{i}\left(t\right)-{\hat{\omega }}_{i}\left(t\right)$.

**Remark** **4.** *In [29], the problems of designing distributed fault-tolerant observers for nonlinear systems with actuator faults and sensor faults are solved through estimating system states and actuator faults. The estimation errors satisfy the uniformly bounded condition, not in asymptotic convergent rate. Therefore, there exists a gap between the estimation and real trajectory. In our paper, based on estimate error of UI-free part ${\tilde{z}}_{i}=z_{i}-{\hat{z}}_{i}$, we can design an observer gains $K_{i}$ and a positive scalar c to guarantee the matrix in (12) is a Hurwitz matrix which means that state observer error dynamic system (12) is asymptotically stable. Additionally, M can be selected as $M=-aI_{q}$, where a>0 denotes the convergence rate, to guarantee that the interval observer error dynamics (17) are bounded stable. Thus, (8) and (24) can provide the theoretically accurate observation.*

## 4. Simulation

We present two numerical simulations in this section.

### 4.1. Example 1

The considered system is in form (1). The matrices of the state equation are $A=I_{4}⊗\left[\begin{array}{cc} -2 & 1\\ 0 & -2 \end{array}\right]$, $B=1_{8}$, $D=1_{4}⊗\mathrm{col}\left(1,0\right)$. Suppose the system is equipped with four sensor groups, where the four output matrices corresponding to the four output equations are

$$C_{1}=\left[\begin{array}{cccccccc} 1 & 0 & 1 & 0 & 0 & 0 & 1 & 0\\ 0 & 1 & 0 & 0 & 0 & 1 & 0 & 0\\ 0 & 0 & 0 & 1 & 0 & 0 & 0 & 0 \end{array}\right],C_{2}=\left[\begin{array}{cccccccc} 0 & 0 & 0 & 1 & 1 & 0 & 0 & 0\\ 0 & 0 & 1 & 0 & 0 & 1 & 0 & 0\\ 0 & 0 & 0 & 0 & 0 & 1 & 0 & 0 \end{array}\right],$$

$$C_{3}=\left[\begin{array}{cccccccc} 0 & 0 & 0 & 0 & 1 & 0 & 0 & 0\\ 0 & 0 & 0 & 0 & 0 & 1 & 0 & 0 \end{array}\right],C_{4}=\left[\begin{array}{cccccccc} 0 & 0 & 0 & 0 & 0 & 1 & 1 & 0\\ 0 & 1 & 0 & 0 & 1 & 0 & 0 & 1\\ 0 & 0 & 0 & 0 & 0 & 1 & 0 & 0 \end{array}\right].$$

Additionally, $W_{1}=\mathrm{col}\left(0,1,1\right)$, $W_{2}=\mathrm{col}\left(1,1,1\right)$, $W_{3}=\mathrm{col}\left(0,1\right)$ and $W_{4}=\mathrm{col}\left(1,0,1\right)$. Moreover, we assume that UI signal impacting on the state equation is $f\left(t\right)=0.25\sin\left(0.5t\right)$, and the MNs are $\omega_{1}=0.2\sin\left(t\right)$, $\omega_{2}=0.25\cos\left(0.5t\right)$, $\omega_{3}=0.4\sin\left(t\right)$ and $\omega_{4}=0.35\cos\left(0.5t\right)$. The input is given as $u=2\cos\left(t\right)$.

**Figure 1:**
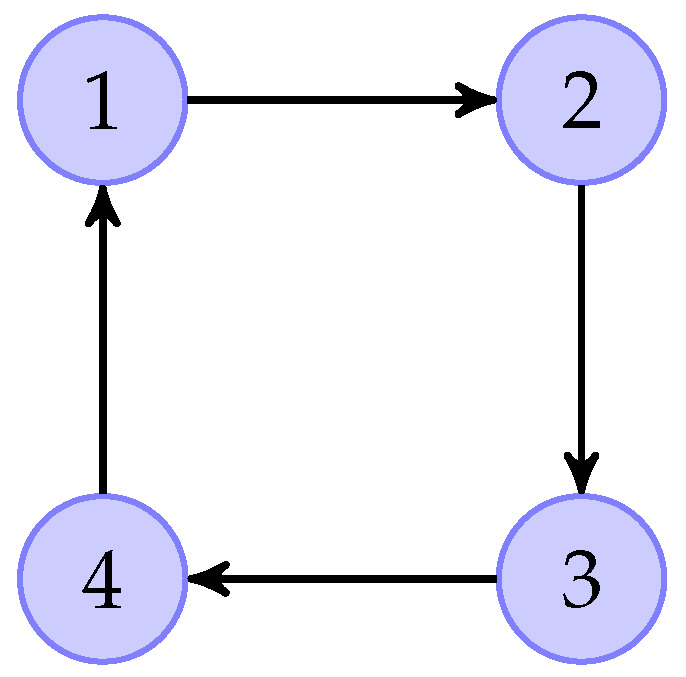
Communication graph topology in Example 1.

The information communication topological graph is shown by Figure 1. Based on Figure 1, we can obtain

$$L=\left[\begin{array}{cccc} 1 & 0 & 0 & -1\\ -1 & 1 & 0 & 0\\ 0 & -1 & 1 & 0\\ 0 & 0 & -1 & 1 \end{array}\right].$$

**Figure 2:**
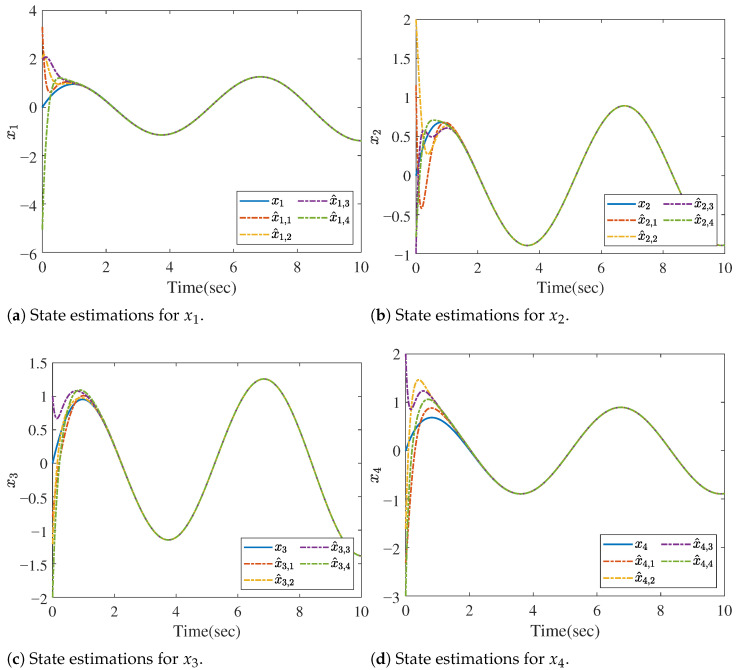
State estimations for $x_{i}$ (i=1,2,3,4).

**Figure 3:**
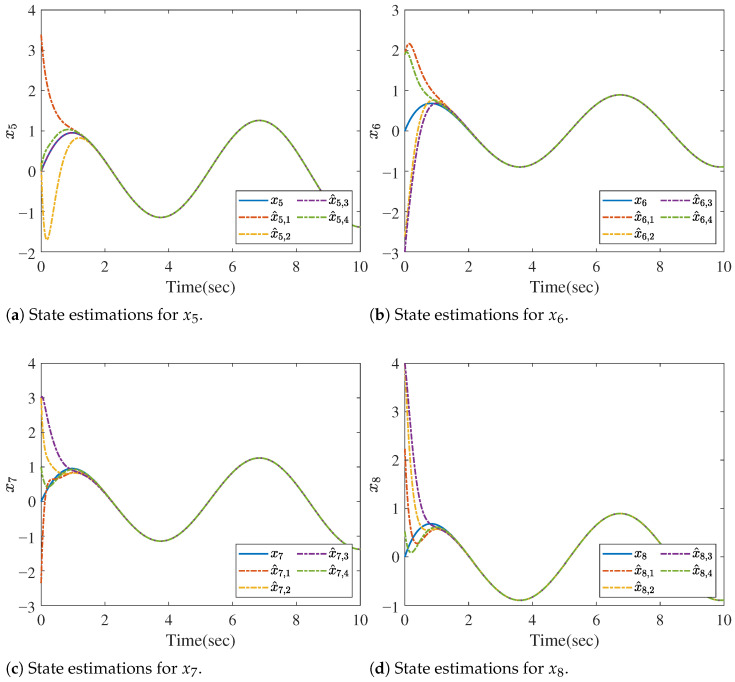
State estimations for $x_{i}$ (i=5,6,7,8).

Since the target system is a 8-dimension dynamic system, we denote the state vector as $x=\mathrm{col}(x_{1},\cdots ,x_{8})$. Now, based on Theorem 1, the asymptotic convergent state estimation can be obtained, and the estimating performances are shown in Figure 2 and Figure 3. Figure 2 offers the state estimations for the state variables $x_{1}$, $x_{2}$, $x_{3}$ and $x_{4}$, while Figure 3 gives the estimations for $x_{5}$, $x_{6}$, $x_{7}$ and $x_{8}$. The notations ${\hat{x}}_{i,j}$ in the figures stand for the estimations of $x_{i}$(i=1,⋯,8) provided by the *j*th(j=1,⋯,4) distributed observer. The initial states are randomly chosen.

**Figure 4:**
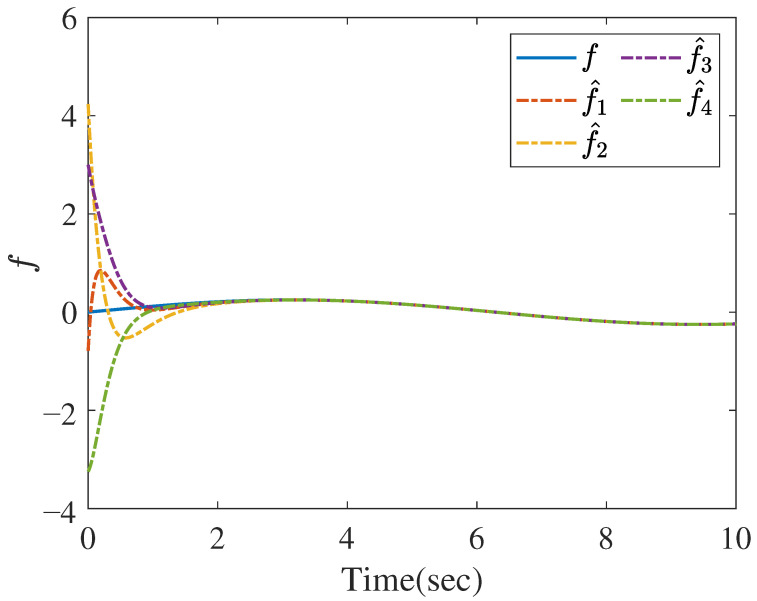
The reconstruction of *f*.

**Figure 5:**
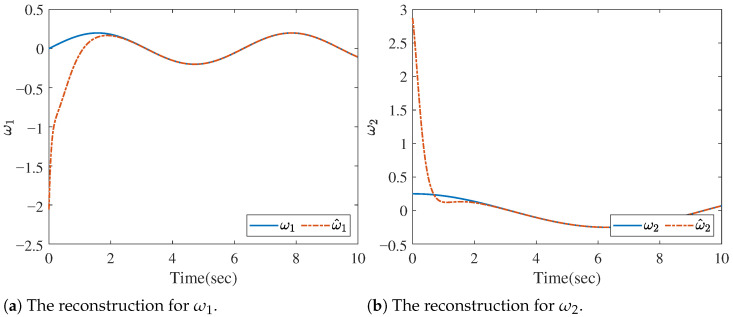

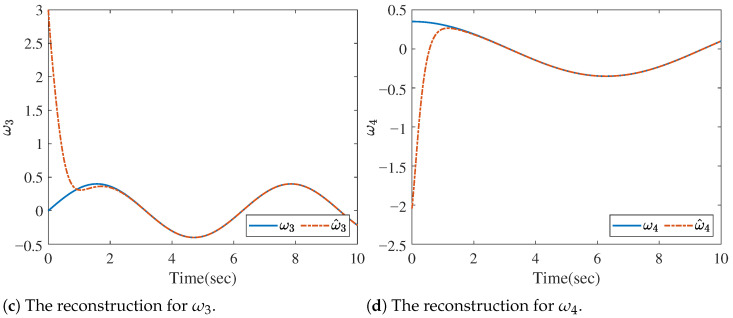
The reconstruction for $\omega_{i}$ (i=1,2,3,4).

**Figure 6:**
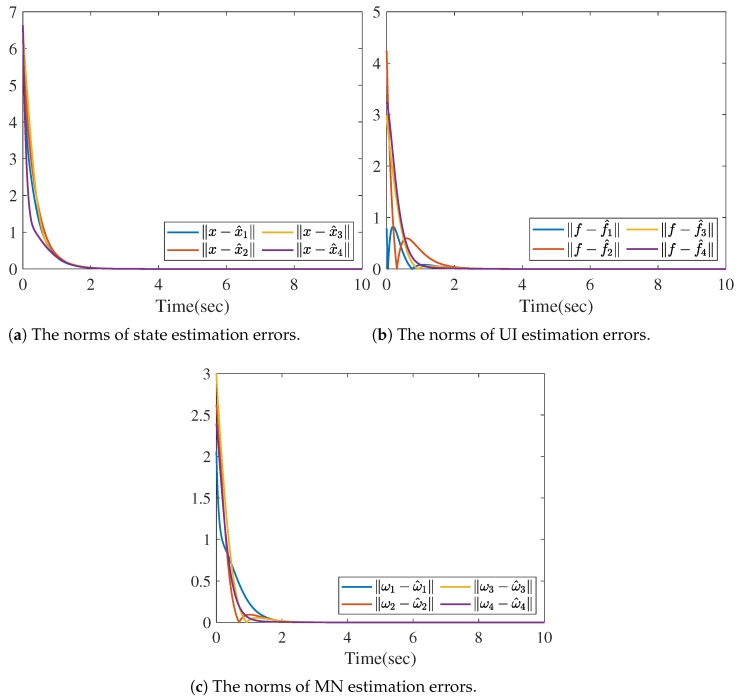
The norms of state, UI, and MN estimation errors.

Figure 4 shows the reconstruction of *f* by (23); that is, $\lim_{t\to \infty }f\left(t\right)-{\hat{f}}_{i}\left(t\right)=0$. Figure 5a–d provide the reconstruction of the measurement noise $\omega_{1}$, $\omega_{2}$, $\omega_{3}$, and $\omega_{4}$, respectively, based on (26). We also present the norms of the state, UI, and MN estimation errors in Figure 6, which clearly show that the errors converge rapidly to zero.

**Figure 7:**
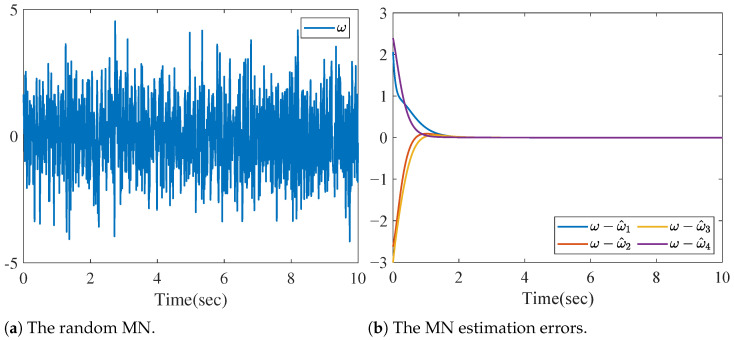
The random MN and MN estimation errors.

To show the effect of the proposed method in more particular case, we consider a random MN $\omega_{i}=\omega$, as shown in Figure 7a. Then, the estimation errors of MN is shown in Figure 7b. It is clear that the dynamic of errors is stable, which validates the effect of the proposed method.

### 4.2. Example 2

**Figure 8:**
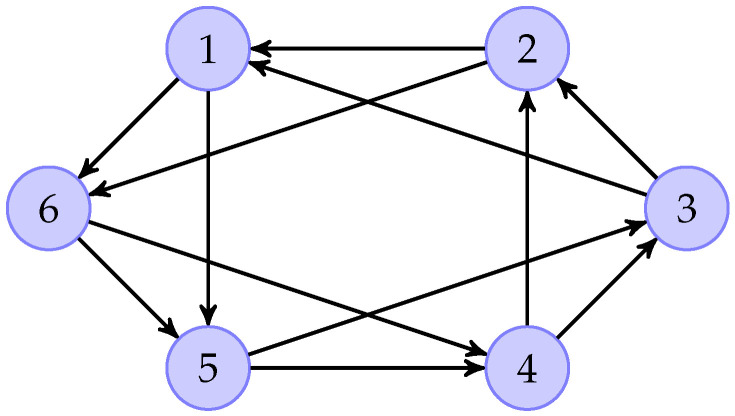
Communication graph topology in Example 2.

Consider an 18-dimension multiple sensor system (1) the matrices of which are $A=I_{6}⊗\left[\begin{array}{ccc} -5 & 0 & 1\\ 0 & -3 & 0\\ 0 & 0 & -2 \end{array}\right],B=D=1_{18}$. Suppose that the system is equipped with six sensors, and the output matrices in of the six sensors are, respectively, $C_{1}=\left[\begin{array}{ccc} I_{2} & I_{2} & 0_{2\times 14} \end{array}\right],C_{2}=\left[\begin{array}{ccc} 0_{4\times 2} & I_{4} & 0_{4\times 12} \end{array}\right],C_{3}=\left[\begin{array}{ccccc} 0_{3\times 6} & I_{3} & 0_{3\times 1} & I_{3} & 0_{3\times 9} \end{array}\right],C_{4}=\left[\begin{array}{ccc} 0_{2\times 9} & I_{2} & 0_{2\times 7} \end{array}\right],C_{5}=\left[\begin{array}{ccc} 0_{3\times 11} & I_{3} & 0_{3\times 4} \end{array}\right],C_{6}=\left[\begin{array}{cc} 0_{4\times 11} & I_{4} \end{array}\right],W_{1}=W4={\left[\begin{array}{cc} 0 & 1 \end{array}\right]}^{T},W_{2}=W_{6}={\left[\begin{array}{cccc} 0 & 0 & 1 & 1 \end{array}\right]}^{T},W_{3}=W_{5}={\left[\begin{array}{ccc} 0 & 1 & 1 \end{array}\right]}^{T}$. The UI is supposed to be f(t)=5sin(2t), and MN are $\omega_{1}=0.1\sin\left(2t\right),\omega_{2}=0.2\cos\left(0.5t\right),\omega_{3}=0.3\sin\left(t\right),\omega_{4}=0.4\cos\left(1.5t\right),\omega_{5}=0.5\sin\left(2t\right);\omega_{6}=0.6\cos\left(0.5t\right);$. The control input is set as u(t)=2cos(0.5t). The initial states are randomly chosen. The information communication topological graph is shown by Figure 8. Additionally, we can obtain the Laplacian matrix as

$$L=\left[\begin{array}{cccccc} 2 & -1 & -1 & 0 & 0 & 0\\ 0 & 2 & -1 & -1 & 0 & 0\\ 0 & 0 & 2 & -1 & -1 & 0\\ 0 & 0 & 0 & 2 & -1 & -1\\ -1 & 0 & 0 & 0 & 2 & -1\\ -1 & -1 & 0 & 0 & 0 & 2 \end{array}\right].$$

**Figure 9:**
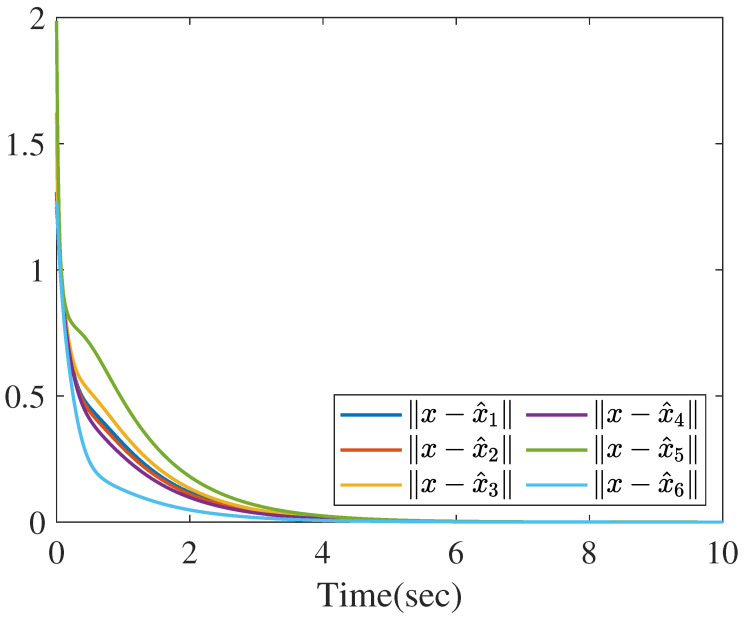
The norms of state estimation errors.

**Figure 10:**
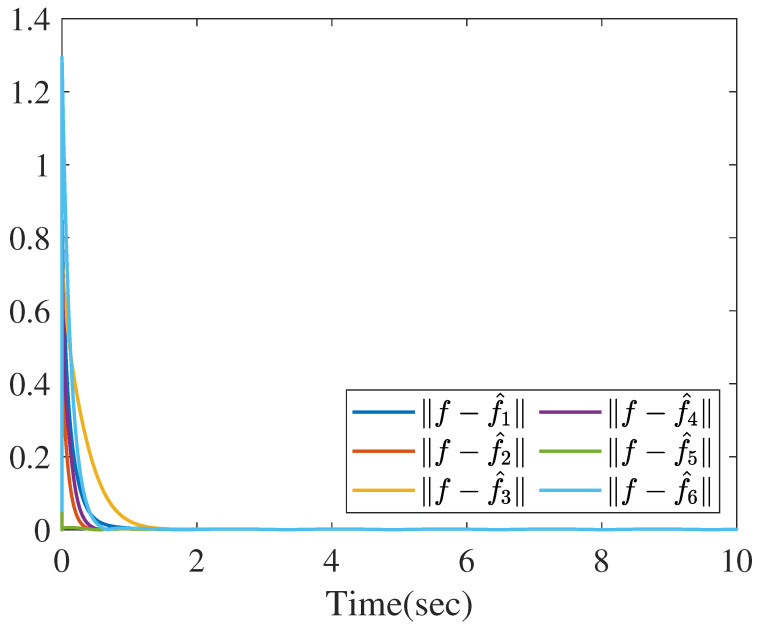
The norms of UI estimation errors.

**Figure 11:**
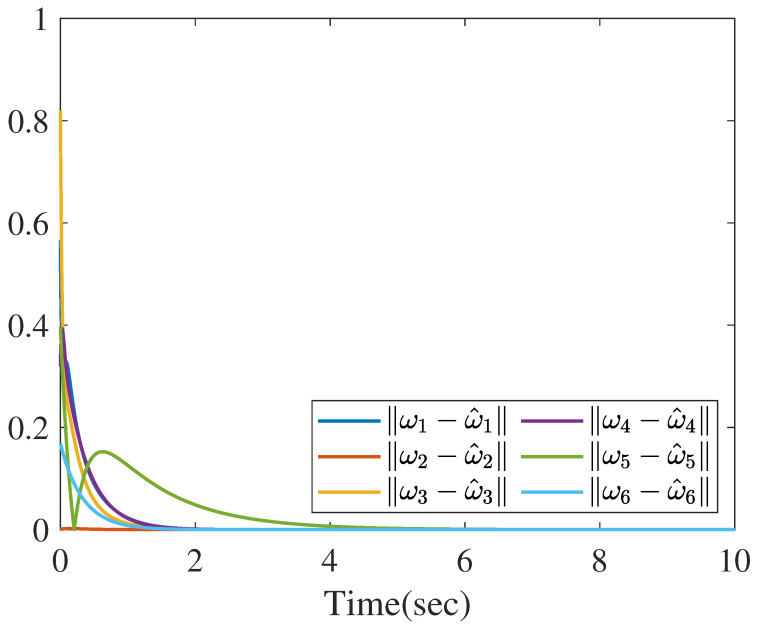
The norms of MN estimation errors.

Based on the distributed reduced-order observer (8), the norms of state estimate errors in all sensors are shown in the Figure 9, which clearly verifies that the proposed distributed observer can generate the asymptotic estimation of state. Furthermore, Figure 10 shows the norms of UI *f* reconstruction errors in all sensors and Figure 11 shows the norms of MN $\omega_{i}$ reconstruction errors in all sensors, which verify that the proposed method can generate the asymptotic estimation of UI and MN, respectively.

## 5. Conclusions

In this paper, for a multiple sensor target system, where the target system is with UI and each sensor node deployed suffers from MN, the design problem of the DUIO is investigated in detail. Through a state equivalent transformation, the original system is decomposed into two constituent parts. One is free from UI and the other is still affected by the UI. For the subsystem being free from UI, a distributed observer is constructed to produce the state estimation. Moreover, by designing an interval observer, we derive an algebraic state-UI relationship, and then a DUIR is provided. Consequently, the DUIO consists of two parts: one is actually a distributed reduced-order observer, and the other is the UIR. Concurrent asymptotic estimation of the UI, system states, and measurement noise is achieved by the proposed reduced-order DUIO. Additionally, the estimation of UI or the UIR decouples the input. How to improve the result to be suitable for the multiple sensor system with switching topology, or sensor data distortion and sensor attacks are slated for future investigation.

## Data Availability

The original contributions presented in this study are included in the article. Further inquiries can be directed to the corresponding author.

## References

[1] Cheng L., Chen X., Zhao F., Qiu J., Cao J. (2025). Observer-based prescribed-time leader-follower consensus for nonlinear multiagent systems under stochastic switching topologies. J. Syst. Sci. Complex..

[2] Zhang L., Guay M., Wang S., Lu M. (2025). Completely distributed state estimation for jointly observable uncertain linear systems. IEEE Trans. Autom. Control.

[3] Koo S., Lee J.G., Shim H. (2025). A distributed observer accommodating a broad range of intermittent communication scenarios. IEEE Control Syst..

[4] Yang Y., Liu F., Yang H., Li Y., Liu Y. (2021). Distributed finite-time integral sliding-mode control for multi-agent systems with multiple disturbances based on nonlinear disturbance observers. J. Syst. Sci. Complex..

[5] Muntean M.V. (2024). Real-Time Detection of IoT Anomalies and Intrusion Data in Smart Cities Using Multi-Agent System. Sensors.

[6] Poh W.Q.T., Naayagi R.T. Modelling and integration of a piezoelectric cantilever beam with quasi-z-source inverter for self-powered dynamic system application. Proceedings of the 2020 IEEE Power & Energy Society General Meeting (PESGM).

[7] Poh W.Q.T., Bin Mohamad Saifuddin M.R., Naayagi R.T. (2020). A novel self-powered dynamic system using a quasi-z-source inverter-based piezoelectric vibration energy harvester. Electronics.

[8] Cai G., Yin G., Liu Y., Feng J., Liang J., Wang F., Liu H. (2025). Stochastic cooperative adaptive cruise control with sensor data distortion and communication delay. IEEE Trans. Intell. Transp..

[9] Chen G., Zhu P., Peng X.-J., Huang C., Li H. (2026). Watermarking-based attack detection for sensor networks with intermittent observation under stealthy attacks. J. Syst. Sci. Complex..

[10] Kim T., Lee C., Shim H. (2020). Completely decentralized design of distributed observer for linear systems. IEEE Trans. Autom. Control.

[11] Ling X., Xu H., Zhu F. (2026). Active compensation fault-tolerant control for uncertain systems with both actuator and sensor faults. Sensors.

[12] Liu T., Wang S., Huang J. (2024). An adaptive distributed observer for a class of uncertain linear leader systems over jointly connected switching networks and its application. IEEE Trans. Autom. Control.

[13] Liu T., Huang J. (2024). Distributed exponential state estimation of linear systems over jointly connected switching networks. Automatica.

[14] Zhang L., Lu M., Deng F., Chen J. (2024). Distributed state estimation under jointly connected switching networks: Continuous-time linear systems and discrete-time linear systems. IEEE Trans. Autom. Control.

[15] Gao Z., Chen K. (2024). Event-triggered fixed-time distributed observers for general linear systems. Knowl.-Based Syst..

[16] Su H., Wu Y., Zheng W.X. (2024). Distributed observer for LTI systems under stochastic impulsive sequential attacks. Automatica.

[17] Li Z., Mo Y. (2024). Secure distributed dynamic state estimation against sparse integrity attack via distributed convex optimization. IEEE Trans. Autom. Control.

[18] Liang L., Su R., Liu S. (2025). Distributed state estimation and fault diagnosis for networked systems under jointly connected switching networks. Automatica.

[19] Liang C., He D., Xu C., Chen Y. (2025). Distributed moving horizon estimation over energy harvesting wireless sensor networks: A switching topology approach. IEEE Trans. Circuits Syst. I Regul..

[20] Wang F., Zhu F. (2024). Distributed State Observer for Systems with Multiple Sensors under Time-Delay Information Exchange. Sensors.

[21] Zhang J., Zhao X., Zheng G., Zhu F., Dinh T.N. (2025). On distributed prescribed-time unknown input observers. IEEE Trans. Autom. Control.

[22] Yang G., Barboni A., Rezaee H., Parisini T. (2022). State estimation using a network of distributed observers with unknown inputs. Automatica.

[23] Cao G., Wang J. (2023). A distributed reduced-order unknown input observer. Automatica.

[24] Cao G., Wang J. (2023). Distributed unknown input observer. IEEE Trans. Autom. Control.

[25] Cacace F., Germani A., Manes C. (2015). A new approach to design interval observers for linear systems. IEEE Trans. Autom. Control.

[26] Chi M., Zhang A., Wang X., Ye L. (2025). Interval observer-based control of Takagi–Sugeno fuzzy systems with uncertainties. IEEE Trans. Fuzzy Syst..

[27] Qian Y., Miao Z., Zhou J., Zhu X. (2025). On consensus control of uncertain multiagent systems based on two types of interval observers. IEEE Trans. Cybern..

[28] Zhu F., Li M. (2024). Distributed interval observer and distributed unknown input observer designs. IEEE Trans. Autom. Control.

[29] Yu C., Su Q., Sun J., Long Y., Zhong G.-X. (2024). Intermediate parameter based distributed sensor fault-tolerant estimation for a class of nonlinear systems. ISA Trans..

[30] Wang X., Su H., Zhang F., Chen G. (2023). A robust distributed interval observer for LTI systems. IEEE Trans. Autom. Control.

[31] Wang X., Xu W., Su H., Gao Z., Chen G. (2024). Designing a completely distributed interval observer for the LTI system. IEEE Trans. Autom. Control.

[32] Huang J., Ma X., Che H., Han Z. (2020). Further result on interval observer design for discrete-time switched systems and application to circuit systems. IEEE Trans. Circuits Syst. II Express Briefs.

[33] Zhu X., Li Y., Yin G., Patton R.J. (2024). Interval observer-based fault detection and isolation for quadrotor UAV with cable-suspended load. IEEE Trans. Syst. Man Cybern. Syst..

[34] Chakrabarty A., Corless M.J., Buzzard G.T., Żak S.H., Rundell A.E. (2017). State and unknown input observers for nonlinear systems with bounded exogenous inputs. IEEE Trans. Autom. Control.

[35] Darouach M., Zasadzinski M., Xu S.J. (1994). Full-order observers for linear systems with unknown inputs. IEEE Trans. Autom. Control.

[36] Corless M., Tu J. (1998). State and input estimation for a class of uncertain systems. Automatica.

[37] Edwards C., Spurgeon S.K., Patton R.J. (2000). Sliding mode observers for fault detection and isolation. Automatica.

[38] Zhu F. (2012). State estimation and unknown input reconstruction via both reduced-order and high-order sliding mode observers. J. Process Control.

[39] Chakrabarty A., Fridman E., Żak S., Buzzard G. (2018). State and unknown input observers for nonlinear systems with delayed measurements. Automatica.

[40] Zhu F., Fu Y., Dinh T.N. (2023). Asymptotic convergence unknown input observer design via interval observer. Automatica.

[41] Mazenc F., Dinh T.N., Niculescu S. (2014). Interval observers for discrete-time systems. Int. J. Robust Nonlinear Control.

[42] Levant A. (2003). Higher-order sliding modes, differentiation and output-feedback control. Int. J. Control.

